# Nandrolone‐induced nuclear accumulation of MyoD protein is mediated by Numb, a Notch inhibitor, in C2C12 myoblasts

**DOI:** 10.14814/phy2.13520

**Published:** 2018-01-15

**Authors:** Xin‐Hua Liu, Rita De Gasperi, William A. Bauman, Christopher P. Cardozo

**Affiliations:** ^1^ National Center for the Medical Consequences of Spinal Cord Injury James J. Peter VA Medical Center Bronx New York; ^2^ Department of Medicine Icahn School of Medicine at Mount Sinai New York New York; ^3^ Department of Psychiatry Icahn School of Medicine at Mount Sinai New York New York; ^4^ Department of Rehabilitation Medicine Icahn School of Medicine at Mount Sinai New York New York; ^5^ Department of Pharmacologic Sciences Icahn School of Medicine at Mount Sinai New York New York

**Keywords:** Androgen receptor, myoblasts, MyoD, nandrolone, Numb

## Abstract

Signaling via the androgen receptor (AR) stimulates myogenic progenitor differentiation. In addition, myogenic differentiation factor D (MyoD) and Numb, a Notch inhibitor, play key roles in regulating myogenic differentiation. Nandrolone, an anabolic steroid, upregulates both MyoD and Numb expression in myogenic cells. However, the molecular mechanisms by which MyoD is upregulated by nandrolone are unclear. Moreover, the potential crosstalk between nandrolone, MyoD, and Numb is not well understood. With these considerations in mind, we examined the effects of nandrolone on the expression of MyoD mRNA and protein, and determined the interactions of MyoD and Numb in the presence or absence of nandrolone in differentiating C2C12 myoblasts. Nandrolone increased MyoD mRNA and protein expression and significantly enhanced nuclear translocation of MyoD protein. The later effect of nandrolone was blunted by siRNA against Numb. Immunoprecipitation (IP) studies confirmed that Numb forms complexes with MyoD. Chromatin IP revealed that in the presence of nandrolone, Numb is recruited to a region of the MyH7 promotor containing the E‐box to which MyoD binds. These data indicate that nandrolone‐regulated MyoD activation occurs mainly through a posttranslational mechanism which promotes MyoD nuclear accumulation, and suggest that this effect of nandrolone is, at least in part, mediated by Numb.

## Introduction

Skeletal muscle fibers are highly adaptable to changes in neuromuscular transmission and atrophy in response to either disuse or denervation (Sandri 2008). MyoD occupies a key role in differentiation of myogenic precursors. It regulates myogenic fate commitment and induces myoblast differentiation. MyoD is a nuclear protein expressed exclusively in skeletal muscle cells and acts as a transcriptional activator of muscle‐specific gene expression during muscle differentiation (Olson 1990; Weintraub et al. 1991). MyoD belongs to the family of muscle‐specific basic helix‐loop‐helix (HLH) proteins. Dimerization between MyoD and HLH proteins, such as the product of E2A gene, is essential for sequence‐specific DNA binding and subsequent transcriptional activation (Murre et al. 1989). Indeed, MyoD has been reported to have a nuclear localization in different cell lines (Tapscott et al. 1988) as well as in MyoD‐transfected cells (Dias et al. 1992), which is in agreement with its transcriptional activity. In addition, two nuclear localization signals (NLSs), one present in the basic region and the other in the helix 1 domain of MyoD protein, have been demonstrated to be functional in promoting the active nuclear transport of MyoD (Vandromme et al. 1995). The nuclear import of MyoD is a rapid and active process, being ATP‐ and temperature‐dependent (Nigg et al. 1991; Vandromme et al. 1995), which has been reported to be mediated by cAMP‐dependent protein kinase activity (Vandromme et al. 1994). Although these reports reveal that MyoD functions as a nuclear transcriptional factor, the question regarding the regulation of differentiation stage‐dependent intracellular localization of MyoD needs to be further investigated.

Androgen signaling via the androgen receptor (AR) is a key pathway that contributes to development, cell fate decision, and differentiation including that of myogenic progenitors (Singh et al. 2003; Sinha‐Hikim et al. 2006). Androgens and anabolic steroids have well‐established pro‐myogenic effects on skeletal muscle development (Taylor et al. 1999; Kicman 2008; Wu et al. 2010). Administration of testosterone promotes the commitment of mesenchymal stem cells to the myogenic lineage, and increases skeletal muscle mass and strength in elderly men and in atrophic states, such as cancer and burn‐induced cachexia (Kicman 2008). Androgens and anabolic steroids have also been demonstrated to reduce muscle loss caused by microgravity (Wimalawansa et al. 1999), immobilization (Taylor et al. 1999), and spinal cord injury (Gregory et al. 2003). The precise mechanisms underlying these androgen actions are, however, poorly understood.

Numb was originally identified for its role in development of the nervous system of flies and for its ability to facilitate asymmetric division of progenitor cells (Spana et al. 1995). More recently, it has been shown to function as an inhibitor of Notch signaling, through targeting Notch and the intracellular domain of Notch receptor (NICD) for proteolytic degradation by the ubiquitin–proteasome pathway (Gulino et al. 2010) and to suppress Shh signaling by targeting Gli for degradation by Itch (Di Marcotullio et al. 2006). Numb has also been shown to promote differentiation of satellite cells of the myogenic lineage (Jory et al. 2009). Deletion of Numb results in stem cells that can proliferate but are unable to generate daughter cells that can differentiate. In satellite cells, knockdown of Numb maintains the cells in the intermediate progenitor stage (Zilian et al. 2001). Conversely, elevated expression of Numb commits satellite cells to a myoblast fate and increases expression of MyoD and Pax7 (Conboy and Rando 2002). Numb protein is highly evolutionarily conserved and displays a complex pattern of functions, such as the control of asymmetric cell division and cell fate determination (Gulino et al. 2010), including controlling endocytosis (Dho et al. 2006). Moreover, Numb protein is now recognized to participate in a number of signaling pathways in addition to Notch and Hedgehog, including p53 and Wnt/*β*‐catenin pathways (Di Marcotullio et al. 2006; Carter and Vousden 2008; Liu et al. 2013).

Nandrolone, an anabolic steroid, has been demonstrated to prevent denervation‐induced atrophy of rat muscle (Zhao et al. 2008a,b). The molecular mechanisms responsible for this effect, however, are not well understood. Nandrolone has been shown to upregulate MyoD expression and activation (Piovesan et al. 2013; MacKrell et al. 2015). More recently, we have reported that nandrolone is able to upregulate Numb protein expression via two complementary mechanisms: stabilizing Numb protein against degradation, presumably by reducing Mdm2 expression (Liu et al. 2012), and upregulating gene expression via activating canonical Wnt/*β*‐catenin signaling (Liu et al. 2013), leading to a reduced Notch signaling (Liu et al. 2011). These data implicate a possible interaction between AR signaling, MyoD and Numb. However, whether such crosstalk exists and how it might be mediated is not well understood. With these considerations in mind, we examined the effects of nandrolone on the expression of MyoD mRNA and protein, and determined the interactions effect of MyoD and Numb in the presence or absence of nandrolone in differentiating C2C12 myoblasts.

## Materials and Methods

### Cell line and cell culture

C2C12 cells were maintained in DMEM containing 10% FBS at 37°C. All experiments were performed with cells that were cultured for 48 h in DMEM containing 2% horse serum (HS) to initiate differentiation. Cells were treated with either 500 nmol/L nandrolone or vehicle as a control for the time period indicated in the figure legend. This dose of nandrolone (500 nmol/L) was selected because it was previously shown to be effective in in vitro (Liu et al. 2012, 2013) and in vivo studies (Zhao et al. 2008a).

### Preparation of cell lysates, immunoprecipitation (IP), and western blotting (WB)

C2C12 cells were lysed, as described previously (26). Briefly, cells were scraped in cold PBS containing 4 mmol/L iodoacetate and lysed in CHAPS buffer (10 mmol/L CHAPS, 2 mmol/L EDTA, pH 8.0, and 4 mmol/L iodoacetate in PBS) with protease inhibitors. Proteins from the cytosolic and nuclear fractions were isolated using a commercial kit from Thermo‐Fisher Sci. (Rockford, IL), according to the manufacturer's instructions. For immunoblotting, cell lysates were electrophoresed on SDS‐polyacrylamide gels, electrophoretically transferred to a polyvinylidene difluoride membrane (Bio‐Rad), and incubated with primary antibodies overnight at 4°C. IP was performed using a kit from Thermo‐Fisher Sci. Antibodies against Numb were purchased from Cell Signaling Technology (Danvers, MA); Anti‐MyoD and *β*‐tubulin antibodies were products of AbCam (Cambridge, MA); Anti‐AR and anti‐histone H1 antibodies were obtained from Santa Cruz Inc. (Santa Cruz, CA).

### Quantitative real‐time PCR

Quantitative real‐time (Rt)‐PCR was performed as described previously (Liu et al. 2013). For each sample, the determinations were performed in triplicate, and the means for the crossing points of triplicates were used in subsequent calculations. mRNA levels were expressed as fold change using the 2^−ΔΔCt^ method (Livak and Schmittgen 2001). Data were normalized relative to 18s RNA.

### siRNA transfection

Small Interfering RNA (siRNA) against Numb and nonsilencing random siRNA (negative control) were purchased from Thermo‐Fisher Sci. Cells were cultured in six‐well plates and transfected with either nonsilencing random siRNA or 20 nmol/L Numb‐siRNA in 100 *μ*L of siRNA transfection reagent (SignaGen, Ijamsville, MD), following the manufacturer's recommended procedures. Cells were then treated with either vehicle or nandrolone under differentiating conditions.

### Chromatin immunoprecipitation (CHIP) assay

A CHIP assay kit (Thermo‐Fisher Sci) was used as described previously (Liu et al. 2013). Briefly, cells were cross‐linked with 1.5% formaldehyde at 37°C for 10 min and collected in lysis buffer on ice. Cell lysates were sonicated and were diluted fivefold with a dilution buffer. Diluted cell lysates (500 *μ*l) were used to immunoprecipitate protein‐DNA complexes. Immunoprecipitated DNA was amplified by PCR (30 cycles) using *Taq* polymerase (New England Biolabs) and then resolved by 1.2% agarose gel electrophoresis. The following primer set was used: forward, 5′‐CAC‐ATT‐TTT‐ATC‐TGG‐TCT‐AAT‐ACA‐CTG‐3′; and reverse, 5′‐ATT‐TTT‐AAT‐AGA‐ACT‐GAA‐AAG‐AGA‐AGT‐CA‐3′.

### Statistics

Data were expressed as means ± SEM. The significance of differences between pairs of means was determined with an unpaired Student's *t*‐test. Statistical calculations were performed with Prism 6.0 (GraphPad).

## Results

### Nandrolone upregulated MyoD mRNA and protein expression

**Figure 1:**
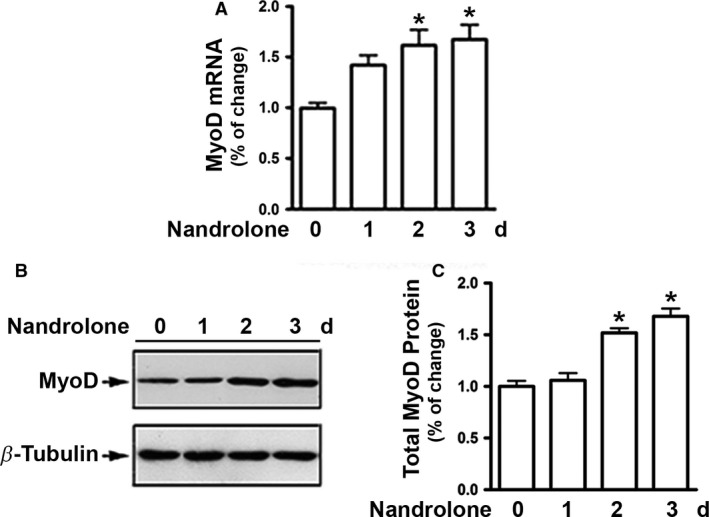
Nandrolone upregulates MyoD mRNA and protein expression. (A)*,* C2C12 cells were cultured in differentiating medium for 48 h then treated with either vehicle or 500 nmol/L nandrolone for various times, as indicated. Total RNA was isolated and subjected to Rt‐PCR analysis. (B)*,* Cells were lysed and total protein was subjected to western blotting. (C)*,* Blots in *B* were quantified by scanning densitometry and normalized relative to *β*‐tubulin. Data shown in *B* are representative western blots. Data shown in *C* are mean values ± SEM for three separate determinations; *P < 0.05.

The effects of nandrolone on MyoD mRNA and protein expression were initially examined on differentiating C2C12 myoblasts. Rt‐PCR revealed that treatment of cells with nandrolone induced a moderate, but significant upregulation in MyoD mRNA which started at 24 h and reached a peak at 48 h after treatment (Fig. 1A). Nandrolone also increased MyoD protein expression with a pattern similar to that seen for mRNA levels (Fig. 1B and C).

### Nandrolone promoted MyoD protein nuclear translocation

**Figure 2:**
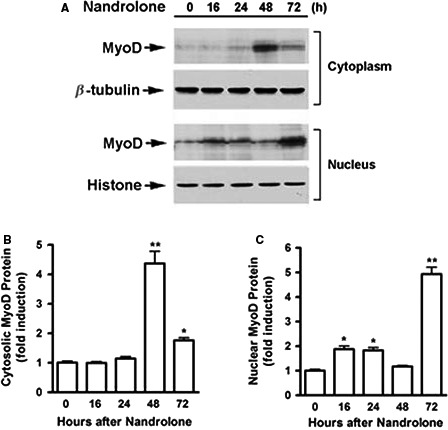
Nandrolone promoted MyoD protein nuclear translocation. (A)*,* C2C12 cells were cultured in differentiating medium for 48 h then treated with either vehicle or 500 nmol/L nandrolone for various times, as indicated. Nuclear and cytosolic proteins were isolated and subjected to western blotting. (B) & (C)*,* Blots in *A* were quantified by scanning densitometry and normalized relative to either *β*‐tubulin (cytosolic protein) or histone H1 (nuclear protein). Data shown in *A* are representative western blots; data shown in B & C are mean values ± SEM for three separate determinations; *P < 0.05, and **P < 0.01.

To evaluate the effect of nandrolone on subcellular distribution of MyoD, we examined MyoD protein expression in cytosolic and the nuclear fractions of C2C12 cells in the presence or absence of nandrolone. WB showed that MyoD levels in the cytosolic fraction were not altered until 48 h after nandrolone addition (Fig. 2A and B). In contrast, we observed an early increase in nuclear MyoD levels, starting at 16 h with a peak at 72 h after treatment with nandrolone (Fig. 2A and C). These results indicated that nandrolone‐induced nuclear accumulation of MyoD was an early event and suggested a role for nandrolone in promoting MyoD nuclear translocation.

### Numb knockdown abolished nandrolone‐induced accumulation of MyoD protein

**Figure 3:**
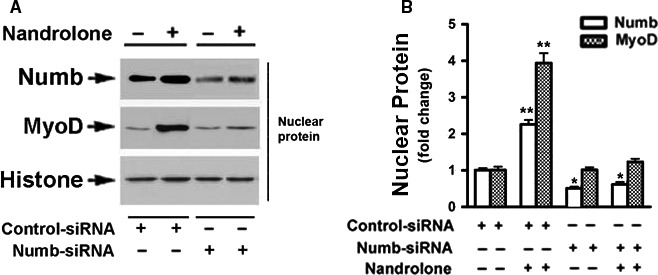
Numb‐siRNA abolished nandrolone‐induced nuclear accumulation of MyoD protein. (A), C2C12 cells were transfected with either nonsilencing random siRNA (negative control) or Numb‐siRNA (20 nmol/L). Cells were then treated with either vehicle or nandrolone (500 nmol/L) for 3 day under differentiating conditions. Nuclear protein was isolated and subjected to western blotting. (B), Blots in A were quantified by scanning densitometry and normalized relative to Histone H1 expression. Data shown in A are representative western blots; data shown in B are mean values ± SEM for three separate determinations; *P < 0.05, and **P < 0.01.

We have reported that nandrolone is able to upregulate Numb gene expression via activating the canonical Wnt/β‐catenin pathway (Liu et al. 2013). To determine whether Numb plays any role in nandrolone‐induced MyoD nuclear translocation, Numb‐siRNA was employed to inhibit the expression of Numb. Cells transfected with Numb‐siRNA demonstrated a significant reduction in Numb protein. Numb knockdown with siRNA did not alter basal levels of MyoD protein, but prevented nandrolone‐induced nuclear accumulation of MyoD protein, compared with the cells transfected with nonsilencing random siRNA (Fig. 3). Thus, Numb was necessary for nuclear translocation of MyoD protein in C2C12 cells. Numb‐siRNA treatment did not affect the 48 h peak in cytosolic MyoD content, as shown in Figure S1, suggesting that nandrolone‐induced upregulation of MyoD mRNA and protein expression is Numb independent.

### Nandrolone promoted the formation of complexes of MyoD with Numb and AR

**Figure 4:**
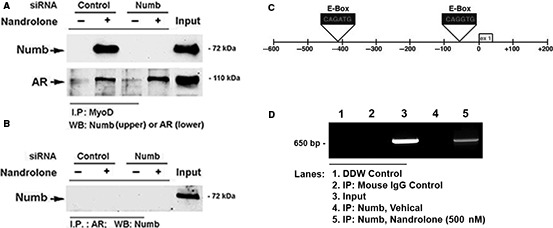
Nandrolone triggered the formation of Numb‐MyoD complexes and promoted the binding of Numb to MyoD binding sites in the promoter region of the slow myosin heavy chain (MyH7) gene. (A) & (B) C2C12 cells were transfected with either nonsilencing random siRNA or Numb‐siRNA (20 nmol/L). Cells were then treated with either vehicle or nandrolone (500 nmol/L) for 3 day under differentiating conditions. Total protein was isolated and subjected to immunoprecipitation (IP) followed by western blotting as indicated. A representative image from two separate experiments is shown. (C) The inset shows a map of the region of the MyH7 promotor containing the E‐Boxes of interest and the PCR strategy used to amplify chromatin pulled down by ChIP. (D) ChIP assay was performed with anti‐Numb antibody and PCR was used to detect the region of the mouse MyH7 promotor containing MyoD binding sites (E‐boxes). A representative image from three independent assays is shown.

We next determined by coimmunoprecipitation whether Numb protein bound to MyoD and/or AR in response to nandrolone treatment. In pull‐downs of the protein complexes formed by either Numb or AR with MyoD using antibody against MyoD, WB confirmed the presence of immunoprecipitated protein of Numb and AR in the presence of nandrolone (Fig. 4A). When Numb protein was knocked down with siRNA, the binding of Numb to MyoD was abolished, whereas the binding of AR with MyoD was not affected (Fig. 4A). In contrast, when pull‐downs from Numb‐siRNA‐transfected cells were conducted with anti‐AR antibody, no Numb protein was observed (Fig. 4B). Thus, MyoD was associated with either Numb or AR in the presence of nandrolone, while Numb was not directly associated with AR suggesting a role of Numb in nandrolone‐induced regulation of MyoD.

### Nandrolone promoted the binding of Numb to the MyH7 promoter

The above findings provided additional evidence supporting a role for Numb in nandrolone‐induced MyoD activation. To test whether Numb might bind MyoD when it is bound at E‐boxes within promoter regions of MyoD target genes, CHIP assays were performed. The assays tested whether exposure of C2C12 cells to nandrolone induced recruitment of Numb to a MyoD binding site of the myosin heavy chain (MyH7) promoter. PCR amplification of DNA fragments derived from vehicle‐treated cells immunoprecipitated with anti‐Numb antibody did not show a band. By contrast, PCR amplification of DNA fragments derived from nandrolone‐treated cells showed a band of 650 bp (Fig. 4D). Sequencing confirmed that this PCR product was amplified from the region of the MyH7 gene containing several known MyoD binding sites (E‐boxes) (Fig. 4C). This finding indicates that Numb binds chromatin in such regions, most likely by tethering to MyoD, and that such binding was promoted by nandrolone.

## Discussion

**Figure 5:**
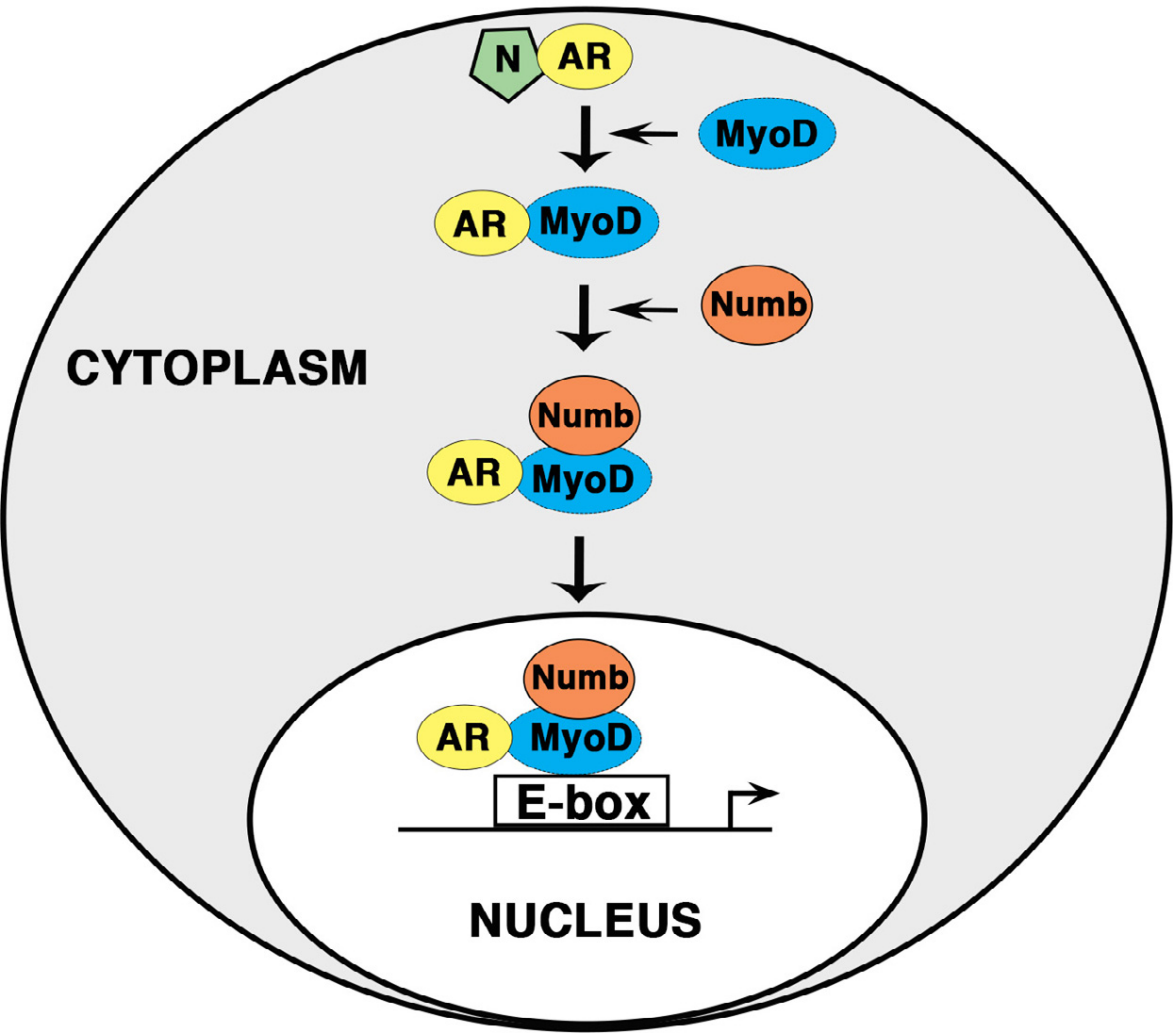
Schema of the effect of nandrolone on the interaction between AR, Numb, MyoD, and gene expression. Androgens stimulate nuclear translocation of MyoD by forming a complex with MyoD. This process requires a direct binding of Numb to MyoD. Moreover, androgens stimulate recruitment of Numb to regions of the MyH7 gene containing known MyoD binding sites to activate MyoD.

This study addressed possible crosstalk between the AR, Numb, and MyoD using differentiating C2C12 mouse myoblasts to gain a greater understanding of the mechanisms by which androgens stimulate differentiation of cells of the myogenic lineage. These studies examined crosstalk stimulated by nandrolone, a synthetic androgen modified to increase its anabolic (e.g., muscle and bone building) activities while minimizing its virilizing actions (hirsutism and gonadal development and function). The results support the following conclusions: androgens stimulate nuclear translocation of MyoD, a critical myogenic differentiation factor; MyoD forms a complex with Numb; androgen‐induced nuclear translocation of MyoD requires Numb; and androgens stimulate recruitment of Numb to regions of the MyH7 gene containing known MyoD binding sites, as summarized in Figure 5. These findings establish for the first time a linkage between androgen action and MyoD, and between MyoD and Numb. The findings suggest that in addition to participating in nuclear accumulation of MyoD, Numb may serve to regulate its transcriptional activity through tethering to chromatin‐bound MyoD thereby altering the repertoire and activity of coregulators bound by MyoD. Moreover, because MyoD drives myogenic differentiation, the findings thus provide novel mechanistic insights regarding the ability of Numb to stimulate myogenic differentiation of progenitors that likely have more broad implications regarding roles of Numb in understanding the role(s) of Numb in satellite cell biology and cell fate commitment. The findings also raise intriguing questions regarding potential roles of Numb in transcriptional regulation of MyoD target genes including whether and how Numb modifies transcription when tethered to MyoD at its cognate DNA binding sites. Further investigation will be required to answer these questions.

We are not aware of other reports linking Numb to nuclear translocation of transcriptional regulators or of other evidence demonstrating that Numb is tethered to chromatin at key regulatory sites in promoter regions or enhancers. However, there is a small but growing number of reports demonstrating that Numb determines the turnover of transcriptional regulators such as Notch1, Gli, and p53 (Frise et al. 1996; Di Marcotullio et al. 2006; Colaluca et al. 2008). For example, Numb forms a tripartate complex with p53 and mdm2 and in so doing stabilizes p53 against ubiquitination and proteasomal degradation (Colaluca et al. 2008). Recently, we observed that nandrolone stabilized Numb protein against degradation by the ubiquitin–proteasome pathway, most likely by reducing Mdm2 protein levels (Liu et al. 2012). Both cytoplasmic and nuclear MyoD is turned over by the ubiquitin–proteasome pathway (Lingbeck et al. 2003) although mechanisms of initial polyubiquitination differ; whereas cytoplasmic MyoD is subject to ubiquitination of lysines, nuclear MyoD appears to undergo both N‐terminal ubiquitination and lysine ubiquitination with N‐terminal ubiquitination playing critical roles in determining rates of degradation (Sadeh et al. 2008). Thus, the possibility exists that Numb may also stabilize nuclear MyoD against nuclear degradation. Alternatively, Numb may facilitate nuclear translocation. Further studies will be required to delineate the respective roles of altered rates of nuclear import and export of MyoD as compared to stabilization of MyoD protein against ubiquitination at its N‐terminus or internal lysines in the net increase in nuclear MyoD levels observed in nandrolone‐treated cells, and to understand the specific mechanisms by which Numb enables such increases.

## Conflict of Interest

None declared

## Data Accessibility

## Supporting information




**Figure S1:** Numb‐siRNA had no effect on nandrolone‐induced upregulation of cytosolic MyoD protein.
