# Supplementary figures and images for: Nandrolone‐induced nuclear accumulation of MyoD protein is mediated by Numb, a Notch inhibitor, in C2C12 myoblasts
Source: Physiol Rep. 2018 Jan 15;6(1):e13520. doi: 10.14814/phy2.13520 (PMC5789652; doi:10.14814/phy2.13520)

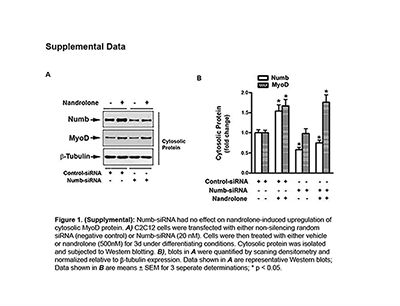

Supplement: Supplementary file 1 — Figure S1: Numb‐siRNA had no effect on nandrolone‐induced upregulation of cytosolic MyoD protein. [file PHY2-6-e13520-s001.tif]
