# Supplementary material for: Nandrolone‐induced nuclear accumulation of MyoD protein is mediated by Numb, a Notch inhibitor, in C2C12 myoblasts
Source: Physiol Rep. 2018 Jan 15;6(1):e13520. doi: 10.14814/phy2.13520 (PMC5789652; doi:10.14814/phy2.13520)
Supplement: Supplementary file 2 [file PHY2-6-e13520-s002.docx]

**Figure Legend**

**Supplemental Data: Figure 1:**

**Figure 1.** (Supplemental): Numb-siRNA had no effect on nandrolone-induced upregulation of cytosolic MyoD protein. ***A)*** C2C12 cells were transfected with either non-silencing random siRNA (negative control) or Numb-siRNA (20 μM). Cells were then treated with either vehicle or nandrolone (500 nM) for 3d under differentiating conditions. Cytosolic protein was isolated and subjected to Western blotting. ***B)*** blots in ***A*** were quantified by scanning densitometry and normalized relative to β-tubulin expression. Data shown in ***A*** are representative western bolts; data shown in ***B*** are means + SEM for 3 separate determinations, * *p* < 0.05.
